# Retraction: Evaluation of coupling coordination development between digital economy and green finance: Evidence from 30 provinces in China

**DOI:** 10.1371/journal.pone.0362716

**Published:** 2026-11-13

**Authors:** The PLOS One Editors

The *PLOS One* Editors retract this article [1] because it was identified as one of a series of submissions for which there are concerns about potential manipulation of the publication process. These concerns call into question the validity and provenance of the reported results. We regret that the issues were not identified prior to the article’s publication.

In addition, the following concerns were noted:

- Extensive text similarity to and potential paraphrasing of a study [2] by different authors, which is not discussed in [1];
- References 5, 43, and 47 were retracted after [1] was published, and Reference 69 was retracted before [1] was published, but after [1] was submitted.

ZL and XZ did not agree with the retraction. JW, LS, and ET either did not respond directly or could not be reached.

## References

[1] Liu Z, Zhang X, Wang J, Shen L, Tang E. RETRACTED: Evaluation of coupling coordination development between digital economy and green finance: Evidence from 30 provinces in China. PLoS One. 2023;18(10):e0291936. doi: 10.1371/journal.pone.0291936 37831729 PMC10575532

[2] Shu H, Zhan L, Lin X, Zhou X. Coordination measure for coupling system of digital economy and rural logistics: An evidence from China. PLoS One. 2023;18(4):e0281271. doi: 10.1371/journal.pone.0281271 37058517 PMC10104290

